# Identifying and managing treatment resistance early with the integration of a clozapine clinic within an early intervention for psychosis service

**DOI:** 10.1111/eip.13578

**Published:** 2024-05-23

**Authors:** Brian O'Donoghue, Linda Mora, Marie Bismark, Andrew Thompson, Patrick McGorry

**Affiliations:** ^1^ Department of Psychiatry University College Dublin Dublin Ireland; ^2^ Department of Psychiatry St Vincents University Hospital Dublin Ireland; ^3^ Centre for Youth Mental Health University of Melbourne Melbourne Victoria Australia; ^4^ Orygen Parkville Victoria Australia; ^5^ Melbourne School of Population and Global Health University of Melbourne Parkville Victoria Australia; ^6^ Te Whatu Ora Kapiti Coast New Zealand

**Keywords:** clozapine, early Intervention, psychosis, schizophrenia, treatment resistance

## Abstract

**Background:**

Despite being the most effective antipsychotic medication for treatment‐resistant psychosis, clozapine is often under‐utilized with long delays to initiation.

**Aims:**

This study aimed to determine whether the integration of a clozapine clinic within an early intervention for psychosis service resulted in a change in the rate and time to initiation of clozapine, the number of trials of different antipsychotic medications prior to clozapine, community initiation and discontinuation rates.

**Methods:**

A clozapine clinic was established in the Early Psychosis Prevention and Intervention Centre in Melbourne. This was a pre‐ and post‐evaluation study design. The ‘clozapine clinic’ cohort included those who commenced on clozapine from 01 January 2016 to 30 June 2018.

**Results:**

Prior to the clozapine clinic, 24 young people commenced clozapine over the 30‐month period compared to 36 in the clozapine clinic cohort (RR = 1.30, 95% CI: 0.75–2.28, *p* = .32). In the pre‐clozapine clinic cohort, 4.6% of all those with a first episode of psychosis were commenced on clozapine compared to 6% in the clozapine clinic cohort. Prior to the clozapine clinic, the median time to the commencement of clozapine was 72 weeks (IQR: 38, 87), compared to 53.5 weeks (IQR: 38, 81.5) in the clozapine clinic (*Z* = −0.86, *p* = .393). The mean number of different antipsychotic medications prior to commencing clozapine remained stable at 3.2 (SD ± 1.1) in both cohorts (*t* = −0.20, *p* = .841). There was a lower rate of discontinuation in the clozapine clinic (43.5% vs. 14.7%, HR = 0.30, 95% CI: 0.09–0.98, *p* = .046).

**Conclusions:**

While this study was underpowered and there are limitations to the naturalistic study design, the findings lend support to the integration of a clozapine clinic within early intervention for psychosis services.

## 1 INTRODUCTION

Despite being the most effective antipsychotic medication for treatment‐resistant psychosis (Siskind et al., 2016), clozapine is under‐utilized and there are often long delays to its commencement when it is prescribed (Thien & O'Donoghue, 2019). This is despite compelling evidence that clozapine results in superior outcomes in a broad range of domains, such as remission of positive symptoms (Siskind et al., 2016), reducing relapses and hospitalisations (Land et al., 2017). There are a number of rare adverse effects associated with clozapine that, if not identified and treated appropriately, can have life‐threatening consequences such as myocarditis (Siskind et al., 2020), constipation leading to bowel obstruction (Hibbard et al., 2009) and agranulocytosis (Li et al., 2020). However, despite these potential adverse effects, clozapine use is associated with a reduction in the early mortality observed in individuals with a diagnosis of schizophrenia (Vermeulen et al., 2019).

At least one‐quarter of individuals with a first episode of psychosis (FEP) will not respond to two adequate trials of antipsychotic medications, thereby meeting criteria for treatment resistance (Siskind et al., 2022). However, it is estimated that at most half of eligible individuals with treatment‐resistant schizophrenia will have a trial of clozapine (Beck et al., 2019) and when it is commenced, delays of over 5 years have been observed in some centres (Thien & O'Donoghue, 2019). In several studies, it was found that individuals tended to have an average of over five trials of different antipsychotic medications before commencing clozapine (Thien & O'Donoghue, 2019). The barriers to commencing clozapine are multi‐factorial and relate to the individual, clinician and service level factors (Thien & O'Donoghue, 2019). The side‐effect profile of clozapine and the need for frequent haematological monitoring has been cited by both individuals and clinicians as a reason for not commencing the medication (Oloyede et al., 2023), although a survey of service users indicated that it would not preclude them from having a trial (Gee et al., 2017). A significant proportion of psychiatrists reported that they prefer to use either high doses of antipsychotic medication or polypharmacy instead of commencing clozapine (Tungaraza & Farooq, 2015), despite the lack of evidence for this strategy and the associated potential adverse effects.

As treatment resistance is identified after there has been an insufficient response to two different antipsychotic medications at an adequate dose for at least 6–8 weeks (Howes et al., 2017), it can be identified within 3–4 months whether an individual with a psychotic disorder is eligible to have a trial of clozapine. Therefore, early intervention for psychosis services are an ideal setting to initiate clozapine and manage the associated side‐effects. In the Early Psychosis Prevention and Intervention Centre (EPPIC) in Melbourne, the proportion of people with a FEP who will subsequently meet criteria for treatment‐resistant schizophrenia has remained relatively stable at just under 10% (Edwards et al., 1998; Thien et al., 2018). In early 2016, a clozapine clinic was established within EPPIC. The service included a dedicated clozapine nurse, a consultant psychiatrist with expertise in clozapine, and access to dietetics and other physical health interventions.

### 1.1 Aims

The aims of the study are to determine whether the introduction of a clozapine clinic within an early intervention for psychosis service resulted in a change in: (i) the rate of initiation of clozapine; (ii) the time to the initiation of clozapine and (iii) the number of trials of different antipsychotic medications prior to the initiation of clozapine; (iv) the rate of initiation of clozapine in the community and (v) discontinuation rates.

## 2 METHODOLOGY

### 2.1 Setting

The EPPIC is an early intervention for psychosis service with Orygen, a youth mental health service that provides care to people aged 15–24 living within the catchment area within northwestern Melbourne. The EPPIC service provides care to approximately 200 people presenting with a FEP annually (Eaton et al., 2019). There is a tenure of care for up to 2 years and this can be extended until the young person reaches the age of 18 if required.

### 2.2 Participants

This study included young people who met the criteria for treatment‐resistant psychosis by not responding appropriately to two trials of different antipsychotic medications, who were commenced on clozapine.

### 2.3 Study design

This was a pre‐ and post‐evaluation study design. The clozapine service was introduced at the start of 2016, and we collected data for a two and half year period following the establishment of the service. Therefore, any individual who commenced on clozapine between 1 January 2016 and 30 June 2018 was included in the ‘clozapine service cohort’, and a corresponding period of 1 January 2013 and 30 June 2015 was used as the comparator, and this group was termed the ‘pre‐clozapine service’ cohort.

### 2.4 Identification of treatment resistance

In EPPIC, young people with persistent positive psychotic symptoms for at least 12 weeks were discussed at the Treatment Resistance Early Assessment Team (TREAT) panel. This consultation panel identifies young people who have not responded to their initial treatment and provide recommendations on pharmacological, psychological and social interventions that may be of benefit to the individual and their family. The TREAT panel also identifies whether an individual would be eligible for clozapine treatment. The definition of treatment‐resistant schizophrenia was that an individual did not respond to two trials of different antipsychotic medications that were of adequate duration (at least 6 weeks) and of adequate therapeutic dose.

### 2.5 Description of the clozapine clinic

The EPPIC service is run across two main clinical sites, with additional satellite clinics across the service. The clozapine clinic was set up in the main clinical sites and each clinic was staffed by a consultant psychiatrist with expertise in clozapine and a clozapine nurse. It was also ensured that the dietician and exercise physiologist were both onsite at the time that the clozapine clinic was running. They could offer individual consultations to the young people either before or after their medical appointment. The young person would typically attend for a blood test at a phlebotomy service in the morning or the day before their medical appointment, and the clozapine nurse would obtain this result prior to the consultation. A physical health exercise group, such as a circuit training, was held prior to the commencement of the clinic and all young people attending the clozapine clinic were encouraged to attend. Therefore, in the service, young people prescribed clozapine were typically seen by a consultant psychiatrist, clozapine nurse and a member of the physical health service (dietician or exercise physiologist). Young people attending the clozapine clinic were also provided with information on smoking cessation, if applicable and nicotine replacement therapy (NRT) was provided (inhaler) or prescribed (NRT patches) if requested.

### 2.6 Sources of information

All of the information obtained in the study was recorded prospectively by clinical staff and collected retrospectively by researchers. Data were collected from registration forms, clinical notes, admission, and discharge summaries.

### 2.7 Statistical analysis

Data were inspected to determine whether it was parametric or non‐parametric and tests of skewness were also applied. Depending upon the distribution of data, the appropriate descriptive test and statistical test were then used, for example, mean and *t*‐tests for parametric data and medians and Mann–Whitney U test for non‐parametric continuous variables.

For the study's first aim, which was to determine whether the initiation of clozapine increased with the introduction of the clozapine clinic, we first needed to determine the denominator or at‐risk population. We obtained information on the total number of new FEP cases that presented to the EPPIC service in the first year of each study period, and this was multiplied by 2.5 to get an estimate of the total number of cases in each study period (as each study period was 2.5 years in duration). We also examined the proportion of people who commenced clozapine from the total cohort of people with a FEP as an indicator of the rate of initiation of clozapine. We had previously identified that 9.4% of young people attending EPPIC would meet the criteria for treatment‐resistant schizophrenia (TRS) (Thien et al., 2018). Cox regression analysis was used to determine hazard ratios for the rate of discontinuation in the two cohorts, as there was a variable time for follow‐up in the different participants.

### 2.8 Power calculation

Our previous study identified that 58% of young people with treatment‐resistant schizophrenia were commenced on clozapine and a realistic target would be for 75% to initiate clozapine. Therefore, a study that could detect this 17% increase in prescribing of clozapine, with 80% power at the 0.05 significant level, would require a total of 240 young people with TRS (120 in the pre‐clozapine clinic cohort and 120 in the clozapine clinic cohort). At EPPIC, it has been demonstrated that <10% of young people will meet criteria for TRS and the meta‐analysis found this figure to be approximately 25%. As a result, it would require a cohort of at least 960 and potentially up 2400 people with a FEP to identify 240 people with TRS, which could take over 10 years to complete. It was deemed that a description of the clinical service and the findings would be of interest to other early intervention services planning on introducing a clozapine clinic, even if underpowered. In addition, the baseline proportion of over half of people with TRS initiating clozapine at EPPIC is likely much higher than other services, as there was already a culture of identifying treatment resistance early. Therefore, services that have a lower baseline proportion of initiating clozapine would require much less numbers to demonstrate a difference, for example, if a service initiated clozapine in 25% of cases of TRS, then a total sample size of 30 would be required.

### 2.9 Ethical approval

*The authors assert that all procedures contributing to this work comply with the ethical standards of the relevant national and institutional committees on human experimentation and with the Helsinki Declaration of 1975, as revised in 2008*. This study was approved by the Melbourne Health Research Ethics Committee as a quality assurance project (QA2017102). As this was a service‐level evaluation, a waiver for individual consent was granted.

## 3 RESULTS

### 3.1 Description of participants

Sixty young people participated in this study, of whom 70% (*n* = 42) were male and the mean age was 19.5 years (SD ± 2.7). A total of 81.7% (*n* = 49) had a diagnosis of treatment‐resistant schizophrenia, while 16.7% (*n* = 10) had a diagnosis of schizoaffective disorder and 1.7% (*n* = 1) had a diagnosis of delusional disorder. The demographic and clinical characteristics of the cohort are presented in Table 1. There were no differences in age, sex and diagnosis between those in the pre‐clozapine clinic cohort and those in the clozapine clinic cohort.

**TABLE 1:** Demographic and clinical characteristics of total cohort, pre‐clozapine clinic and clozapine clinic cohorts.

	Total cohort (*N* = 60)		Pre‐clozapine clinic cohort (*N* = 24)		Clozapine clinic cohort (*N* = 36)		Statistical test of difference	*p*
	Mean	SD	Mean	SD	Mean	SD	*t*, df	
Age	19.5	2.7	19.1	2.2	19.8	3.0	−0.95, 57	.346

	%	*N*	%	*N*	%	*N*	*Χ* ^2^, df	
Sex								.908
Male	70.0	42	70.8	17	69.4	25	0.01, 1	
Female	30.0	18	29.2%	7	30.6	11		
Diagnosis								
Schizophrenia	81.7	49	75.0	18	86.1	31	2.55, 2	.279
Schizoaffective disorder	16.7	10	25.0	6	11.1	4		
Delusional disorder	1.7	1	0	0	2.8	1		

### 3.2 Rates of initiation of clozapine

In the ‘pre‐clozapine clinic’ cohort, 24 young people were commenced on clozapine over the 30‐month period, and an estimated 520 people presented with a FEP during this study period. A total of 36 young people were commenced on clozapine during the first 30 months of the clozapine clinic, and an estimated 600 people presented with a FEP during this time. The rate ratio of commencing clozapine between the two study periods was 1.30 (95% CI 0.75–2.28, *p* = .32).

In regard to the proportion of young people who commenced clozapine, in the pre‐clozapine clinic cohort, 4.6% of the young people with an FEP were commenced on clozapine, and this represented 48.9% of the estimated target of 9.4% that have been previously determined to meet criteria for treatment resistance. In the clozapine clinic, 6% of all those with a FEP were commenced on clozapine, representing 63.8% of those estimated to meet the criteria for treatment resistance. The rates of initiation of clozapine in both cohorts are presented in Table 2.

**TABLE 2:** Initiation, highest dose, and discontinuation of clozapine in both cohorts.

	Pre‐clozapine clinic cohort		Clozapine clinic cohort		Statistical test of difference	*p*
Initiation	%	*N*	%	*N*	RR (95% CI)	
Total estimated number of FEP cases	100	520	100	600	‐	
Initiation of clozapine	4.6	24	6.0	36	1.30 (0.75–2.28)	.320
Time to initiation of clozapine						
Median number of weeks (IQR)	72.0	38–87	53.5	38–81.5	−0.86	.393
Mean number of weeks (SD)	64.9	34.0	60.1	27.2	0.60, 57	.550

	Mean	SD	Mean	SD	*t*, df	
Number of trials of antipsychotic medications prior to clozapine	3.2	1.05	3.2	1.05	−0.20, 58	.841

	%	*N*	%	*N*		
Two	37.5	9	27.8	10		
Three	16.7	4	36.1	13		
Four	37.5	9	25.0	9		
Five	8.3	2	8.3	3		
Six	0	0	2.8	1		

Location of initiation of clozapine	%	*N*	%	*N*	OR (95% CI)	
Community	26.1	6	38.9	14	0.56 (0.18–1.75)	.314
Inpatient	73.9	17	61.1	39		

Dose of clozapine	Mean	SD	Mean	SD	*t*‐test, df	
Mean highest dose of clozapine	315	141	375	113	−1.78, 56	.081

Discontinuation	%	*N*	%	*N*	HR (95% CI)	
Number of young people who discontinued clozapine	43.5	10	14.7	5	0.30 (0.09–0.98)	.046
Reason for discontinuation						
Missing data	‐	0	40.0	2		
Myocarditis	20.0	2	20.0	1		
Other cardiac complications	20.0	2	0	0		
Neutropenia	20.0	2	0	0		
GI side‐effects–constipation	10.0	1	0	0		
Non‐compliance	20.0	2	20.0	1		
At the request of patient	10.0	1	20.0	1		

### 3.3 Time to the initiation of clozapine

In the pre‐clozapine clinic cohort, there was data available for 95.8% (*n* = 23) of participants and the median time to the initiation of clozapine was 72 weeks (IQR 38, 87). In the clozapine clinic cohort (*n* = 36), the median time to the initiation of clozapine was 53.5 weeks (IQR 38, 81.5). There was no statistical difference between the time to initiation of clozapine between the two cohorts (*Z* = ‐0.86, *p* = .393) **(**Table 2 **)**.

### 3.4 Number of trials of different antipsychotic medications prior to the initiation of clozapine

The mean number of trials of different antipsychotic medications prior to the commencement of clozapine was 3.2 (SD ± 1.05) in both cohorts (*t* = −0.20, *p* = .841). In the pre‐clozapine clinic cohort, 37.5% (*n* = 9) of individuals initiated clozapine after two trials of antipsychotic medications compared to 27.8% (*n* = 10) in the clozapine clinic cohort **(**Table 2 **)**.

### 3.5 Community initiation of clozapine

Prior to the clozapine clinic, 26.1% (*n* = 6) of young people initiated clozapine in the community, that is, they were not admitted to hospital, compared to 38.9% (*n* = 14) after the clozapine clinic was established (OR = 0.56, 95% CI: 0.18–1.75, *p* = .312) **(**Table 2 **)**.

### 3.6 Highest dose of clozapine

In the pre‐clozapine clinic cohort, the mean dose of clozapine prescribed was 315 mg (±141) and in the clozapine clinic cohort it was 375 mg (±113) (*t* = −1.78, df = 56, *p* = .081) **(**Table 2 **)**.

### 3.7 Rates of discontinuation

There was information available on rates of discontinuation for 23 in the per‐clozapine clinic cohort and 34 in the clozapine clinic cohort. There was a lower rate of discontinuation of clozapine in the clozapine cohort, with 43.5% (*n* = 10) of those in the pre‐clozapine clinic cohort discontinuing clozapine compared to 14.7% (*n* = 5) in the clozapine clinic (HR = 0.30, 95% CI 0.09–0.98, *p* = .046). However, one young person in the pre‐clozapine clinic cohort discontinued due to neutropenia but had a subsequent trial of clozapine, which was tolerated and was not discontinued at the last observation. The reasons for discontinuation in each cohort are presented in Table 2.

## 4 DISCUSSION

### 4.1 Summary of findings

The main findings of this study are that following the establishment of a clozapine clinic with an EIS, there were absolute increases in the rate and proportion of those with a FEP commencing clozapine and a reduction in the median time to commencement by approximately 4 months, although these differences were not statistically significant. The mean number of trials of different antipsychotic medications that were trialled before initiating clozapine remained the same at three. There was also a lower rate of discontinuation of clozapine after the clozapine clinic was established.

### 4.2 Explanation for findings and clinical implications

The results of this study need to be considered alongside a number of factors. First, while one of the objectives of the clozapine clinic was to reduce delays to the initiation of clozapine in eligible individuals, it is possible that the increased awareness of the effectiveness of clozapine and the streamlined system to provide ongoing monitoring led to some individuals with a longer history of treatment resistance being identified and commencing clozapine. Therefore, it could take a number of years to observe the true reduction in the time to initiation. Second, it needs to be considered that there was already an established practice of trying to identify treatment resistance in a timely manner within the EPPIC service, as a consultation‐based service called the TREAT was established in the EPPIC over 25 years ago (Edwards et al., 2002). Therefore, it is possible that the establishment of a clozapine clinic in other EIS without a historical focus on treatment resistance could have a more profound impact on the proportion commencing clozapine and the time to initiation.

One of the audit standards for early intervention for psychosis services in the United Kingdom and Ireland is the proportion of people with treatment resistance who initiate or are offered clozapine. In the United Kingdom, just less than half of individuals with treatment resistance initiate or are offered clozapine (NCAP, 2022), although the definition applied to treatment resistance in this audit is when an individual does not respond or tolerate two antipsychotic medications, while the consensus definition of treatment resistance does not count a trial that is discontinued based on tolerability (Howes et al., 2017) and therefore this method by the national audit would overestimate the prevalence of treatment resistance.

A limited number of demographic and clinical factors have been found to be associated with a better outcome with clozapine and they are a younger age at commencement, less negative symptoms and the paranoid sub‐type of schizophrenia (Okhuijsen‐Pfeifer et al., 2020). Additionally, it has been found that earlier use of clozapine is associated with better outcomes, specifically for negative symptoms and functioning (Muñoz‐Manchado et al., 2023). However, in this study by Munoz‐Manchado et al, the ‘early’ use of clozapine was defined as initiation within 3 years of the FEP. Considering that an adequate trial of an antipsychotic medication takes between 6 and 8 weeks, this means that treatment resistance can be identified within 12–16 weeks (Howes et al., 2017). Even allowing time for a trial of antipsychotic medication that is discontinued due to tolerability or a longer time for a trial of a long acting injectable, cases of treatment resistance from illness onset should be identified within 6 months of first presentation. Therefore, it is likely that the perception that the initiation of clozapine within 3 years constitutes ‘early use’ stems from the very long delays in the initiation of clozapine within clinical practice. In a recent meta‐analysis that is currently under review, it was demonstrated that after two ineffective trials of antipsychotic medications, the response rate to clozapine was 45% compared to 15% if a third antipsychotic (not clozapine) was trialled (O'Donoghue et al., 2024). Systems or infrastructure, such as a clinical quality registry (Thompson et al., 2023) that can routinely assess symptoms would assist in the identification of prolonged symptoms and treatment resistance. Therefore, the identification of treatment resistance and initiation/offering of clozapine within 6 months would be a reasonable initial target to maximize rates of recovery for individuals with treatment‐resistant schizophrenia. After this has been achieved, there would be further merit in trying to identify individuals who are more likely to respond to clozapine even earlier. The finding that there was a lower rate of discontinuation of clozapine is intriguing and encouraging. While the study design does not allow us to determine the reasons for the lower rate of discontinuation, it may be the that structure of the clozapine clinic and the proactive management of common side‐effects led to better tolerance and adherence. Furthermore, while not a significant difference, there was an absolute lower proportion of people who were admitted to hospital to initiate clozapine following the establishment of the clozapine clinic and if this was replicated in a larger study, it could represent considerable cost savings and also reduce the burden for people initiating clozapine.

### 4.3 Study limitations

The findings of this study need to be considered alongside its limitations. First, the study was not sufficiently powered to detect a statistical difference in the rates of initiation of clozapine in the two different periods. This study was located within a very large early intervention for psychosis service but as the proportion with treatment resistance is relatively low (approximately 10%), the numbers eligible and initiation clozapine are low. Second, we did not have data in relation to the reason as to why individuals discontinued the prior trials of antipsychotic medications and it is likely that some of these trials were discontinued due to tolerability issues.

### 4.4 Conclusions

While this study was underpowered and there are limitations to the naturalistic study design, there are some important findings from this study in relation to the integration of a clozapine clinic within early intervention for psychosis services. There were improvements in two of the key outcomes, namely the rate of clozapine initiation and a reduction in the time to commencement, albeit these were not statistically significant as it would require a larger sample to be sufficiently powered. These result support the identification of treatment resistance and the initiation of clozapine within early intervention for psychosis services.

## CONFLICT OF INTEREST STATEMENT

The authors have no conflicts of interest to declare.

## FUNDING INFORMATION

This study did not receive any specific funding.

## Data Availability

The data that support the findings of this study are available on request from the corresponding author. The data are not publicly available due to privacy or ethical restrictions.
